# Bimetallic Cu-Bi catalysts for efficient electroreduction of CO_2_ to formate

**DOI:** 10.3389/fchem.2022.983778

**Published:** 2022-10-03

**Authors:** Le Li, Xuan Jin, Xiaohan Yu, Miao Zhong

**Affiliations:** National Laboratory of Solid State Microstructures, Jiangsu Key Laboratory of Artificial Functional Materials, College of Engineering and Applied Sciences, Nanjing University, Nanjing, China

**Keywords:** electrocatalysis, Cu-Bi, bimetal, CO_2_R, formate

## Abstract

Electrochemical CO_2_ reduction offers an effective means to store renewable electricity in value-added chemical feedstocks. Much effort has been made to develop catalysts that achieve high Faradaic efficiency toward Formate production, but the catalysts still need high operating potentials to drive the CO_2_–to–formate reduction. Here we report physical vapor deposition to fabricate homogeneously alloyed, compositionally controlled Cu_*1-x*_Bi_*x*_ bimetallic catalysts over a large area with excellent electrical conductivity. Operating electrochemical studies in Ar-saturated and CO_2_-saturated electrolytes identified that Cu–Bi catalysts notably suppress the competing H_2_ evolution reaction and enhance CO_2_–to–formate selectivity. We reported a formate Faradaic efficiency of >95% at an improved cathodic potential of ∼−0.72 V vs. RHE and a high formate cathodic energy efficiency of ∼70%. The electrochemical reaction is stable over 24 h at a current density of 200 mA cm^−2^. The work shows the advantages of bimetallic catalysts over single metal catalysts for increased reaction activity and selectivity.

## Introduction

With the rapid development of modern society, the excessive burning of fossil fuels has led to huge amounts of CO_2_ emissions, breaking the ecological carbon cycle and causing the growing greenhouse effect (Singh et al., 2016; Xiao et al., 2017; Garza et al., 2018; Birdja et al., 2019; Li et al., 2020). Renewably powered CO_2_ conversion to value-added fuels or chemical raw materials, such as CO, HCOO^−^, C_2_H_4_, C_2_H_5_OH, etc., is becoming an important way to maintain energy and environmental sustainability (Kortlever et al., 2015; Wang et al., 2021a; Wang et al., 2021b; Wang et al., 2022). For example, the electroreduction of CO_2_ using renewable electricity has attracted great research attention (Wang et al., 2021c). Among the commonly reported CO_2_R products, formic acid or formate stands out as a promising liquid chemical due to its high energy value in the techno-economic analysis and high volumetric mass density (53.4 g L^−1^) for easy storage and transport (Yoo et al., 2016; Chi et al., 2021).

Conventionally, p block metals such as Bi, Pb, and In have appropriate *OCHO binding energy, favoring the CO_2_–to–formate conversion. However, the electrical conductivity of these metals is not satisfactory, causing a large potential loss at high current densities. Cu is naturally abundant and has good electrical conductivity, possible for practical use (Huang et al., 2018). Cu-based materials are widely investigated as electrocatalysts for CO_2_R to multi-carbon (C_2+_) production (Mistry et al., 2016a; Mistry et al., 2016b; Gao et al., 2019; Zaza et al., 2022). However, due to the modest binding to H, C, and O (Qin et al., 2019; Zheng et al., 2021; Zhang et al., 2022), Cu shows poor selectivity to one specific product which leads to an increased product separation cost.

Bimetallic catalysts have been reported to increase the activity and selectivity of electrocatalytic CO_2_R by taking the advantage of both metals. Zeng et al. improved the formate production by fabricating single atom Pb anchored Cu catalysts (Zheng et al., 2021); Thomas J. Meyer et al. enhanced selectivity for methane production by forming Cu-Pd bimetal alloys (Zhang et al., 2015); Douglas R. MacFarlane et al. improved the electrocatalytic reduction of CO_2_ to CO by loading Au on Cu (Chen et al., 2017); Wang et al. (2010) prepared novel silver-coated nanoporous copper composite electrocatalysts for CO_2_R to produce dimethyl carbonate. As for formate production, Bi is extensively studied as a promising CO_2_R–to–formate catalyst due to its abundance on Earth, low cost, and environmental-benign properties. Jiang and collaborators reported that a Bi nanostructured catalyst electrochemically reduced by BiOCl nanosheets obtained 92% FE_HCOOH_ at −1.5 V vs. SCE at room temperature (Zhang et al., 2014); Zhong et al. achieved over 95% formate selectivity with ultra-long stability of more than 100 days (Li et al., 2021); Li et al. (2019) achieved nearly 100% formate selectivity using Bi/Bi_2_O_3_ with abundant grain boundaries as catalysts. Yet the Bi-based catalysts still require a high potential to conduct electrocatalytic reduction of CO_2_ (Tian et al., 2021).

Herein, we present the large-area fabrication of Cu_*1-x*_Bi_*x*_ (*x* = 0.1, 0.2, 0.25) catalysts using controllable thermal evaporation. We evaluated the CO_2_R and also the competing hydrogen evolution reaction (HER) performance in flow cells in CO_2_-saturated and Ar-saturated electrolytes. We obtained a formate selectivity of 95% and a cathode energy efficiency of 70% at −0.72 V vs. RHE with Cu_0.8_Bi_0.2_. The CO_2_R can proceed stably and efficiently at a current density of 200 mA cm^−2^ over 24 h. We conclude that the bimetallic Cu_0.8_Bi_0.2_ improves formate selectivity and enhances the CO_2_R activity and cathodic energy efficiency, which may offer new perspectives for future design and synthesis of bimetallic CO_2_R catalysts.

## Experimental section

### Synthesis of Cu_*1-x*_Bi_*x*_ (*x* = 0.1, 0.2, 0.25), Cu and Bi catalyst

Cu_*1-x*_Bi_*x*_ (*x* = 0.1, 0.2, 0.25), Cu, and Bi catalysts were synthesized by using the thermal evaporation (SKY-RH400) method. To prepare the Cu_*1-x*_Bi_*x*_ (*x* = 0.1, 0.2, 0.25) catalyst, the precursors Cu and Bi particles are evaporated onto the PTFE gas diffusion electrodes by thermal evaporation. In brief, 2 g of Bi metal particles and 2 g of Cu metal particles were put into the tungsten boats in the deposition chamber. The metal powder was slowly melted in a vacuum environment below 5*10^–4^ Pa. The evaporation rate of Bi was set to 0.03, 0.04, and 0.05 nm s^−1^ and Cu was set to 0.07, 0.06, and 0.05 nm s^−1^ to obtain Cu_*1-x*_Bi_*x*_ (*x* = 0.1, 0.2, 0.25) samples. The thickness of the as-deposited Cu_*1-x*_Bi_*x*_ (*x* = 0.1, 0.2, 0.25) films was about 500 nm as measured by a thickness meter placed inside the evaporation chamber. Uniform bimetallic films were obtained. To prepare the pure Bi and Cu films with the same thickness as control samples, we evaporated both Bi and Cu at 0.1 nm s^−1^ under the same conditions.

### Characterization

Field Emission Scanning Electron Microscope (FESEM) images were taken on a SU8100 Scanning Electron Microscope. SEM-EDX test voltage is 20 kV. Powder X-ray diffraction (XRD) was performed using a Bruker D8 Advance X-ray diffractometer using Cu Kα radiation (*λ* = 0.15418 nm) in the 2θ range of 20°–80° at a scan rate of 7°/min. X-ray photoelectron spectroscopy (XPS) studies were performed using PHI5000 VersaProbe. The binding energy data were calibrated relative to the C 1s signal at 284.6 eV.

### Electrochemical measurements

The electrochemical CO_2_R experiments were carried out in a flow cell setup of a three-electrode system. The CO_2_R catalysts, Ag/AgCl electrodes, and foamed nickel films were used as working electrodes, reference electrodes, and counter electrodes, respectively. 1 M KOH electrolytes were used as both catholyte and anolyte. An anion exchange membrane (Fumasep FAB-PK-130) was used to separate the catholyte and anolyte. All measurements were performed by using an electrochemical workstation (AOTU-Lab). All experiments were performed under the standard conditions with a CO_2_ gas flow rate of 25 standard cubic centimeter per minute (sccm) at the flow cell outlet. The potential range of linear sweep voltammetry (LSV) is 0 to −1.2 V_RHE_, with a sweep speed of 50 mV s^−1^. The electrode potentials were converted to the RHE potentials using V_RHE_ = V_Ag/AgCl_ + 0.197 + 0.059 × pH. Electrochemical impedance spectroscopy (EIS) was measured at open circuit potential with amplitudes of 10 mV over the frequency range of 1 MHz to 1 Hz. The liquid products of CO_2_ reduction were quantitatively analyzed by ion chromatography (Shenghan ICS-1000). Gaseous products were analyzed by gas chromatography (PE GC9790). ^1^H nuclear magnetic resonance (NMR) spectroscopy was used to identify other liquid products other than formate. All measurements were made at room temperature and ambient pressure.

The Faradaic Efficiency (FE) of formate is calculated as follows:

$${FE}_{formate}=\frac{n*F*V*c}{1000*M*Q}$$

where *n* is the number of electrons transferred (*n* = 2). *F* is Faraday’s constant (96,485 C mol^−1^). *c* is the mass concentration of formate produced by the reaction (mg L^−1^). *V* is the volume of catholyte (L). *M* is the molar mass of formate (46.03 g mol^−1^). Q is the total amount of charge consumed by the entire reaction monitored by the electrochemical workstation (C).

The FE of the gas product is calculated according to the following equation:

$${FE}_{gas}=\frac{n*F*V}{1000*22.4*Q}$$

where *n* is the number of electrons transferred (*n* = 2). *F* is Faraday’s constant (96,485 C mol^−1^). *V* is the volume of catholyte (L). *Q* is the total amount of charge consumed by the entire reaction monitored by the electrochemical workstation (C).

Cathode Energy Efficiency (CEE) Calculation Formula:

$${CEE}_{formate}=\frac{\left(1.23-E_{formate}\right)*{FE}_{formate}}{1.23-E_{cathode}}$$

where *E*_*formate*_ of −0.199 V vs. RHE is the standard potential of the formate formation. *FE*_*formate*_ is the measured formate Faradaic efficiency. *E*_*cathode*_ is the applied potential vs. RHE.

## Results and discussion

**FIGURE 1:**
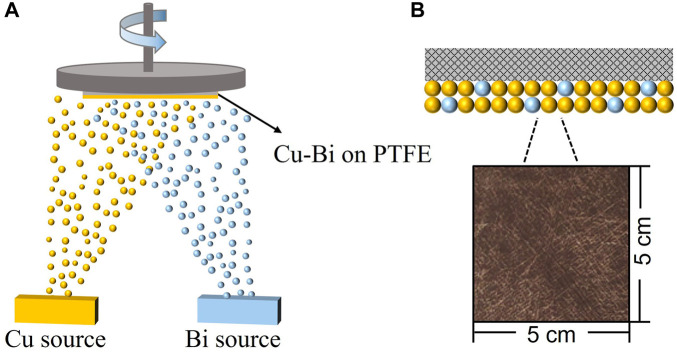
**(A)** A schematic illustration of the synthetic process of Cu-Bi catalyst on polytetrafluoroethylene (PTFE). **(B)** The insert optical image shows a 5 cm^2^ × 5 cm^2^ sample of Cu_0.8_Bi_0.2_ on the PTFE substrate.

**FIGURE 2:**
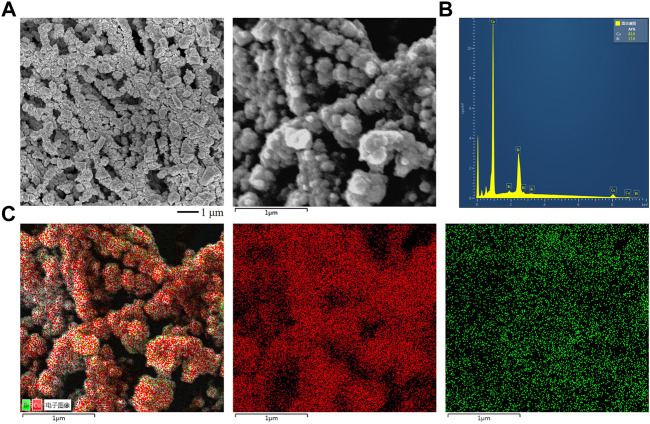
**(A)** SEM images of the synthesized Cu_0.8_Bi_0.2_ by thermal evaporation. **(B)** EDX spectrum of the Cu_0.8_Bi_0.2_. **(C)** EDX Mapping results of the Cu_0.8_Bi_0.2_.

We prepared a series of Cu_*1-x*_Bi_*x*_ (*x* = 0.1, 0.2, 0.25) bimetallic materials, pure Cu, and pure Bi samples on a large area by thermal evaporation. As shown in Figures 1A,B and Supplementary Figure S1, the as-prepared Cu_*1-x*_Bi_*x*_ (*x* = 0.1, 0.2, 0.25) catalysts were all tightly wrapped on the PTFE fibers and formed uniformly distributed Cu and Bi nanoparticles with a size of 100–200 nm. It can be seen from Figure 2A and Supplementary Figure S1 that with the increase of Bi content, the particles gradually form nanocrystals, and the particle size of Cu_*1-x*_Bi_*x*_ (*x* = 0.1, 0.2, 0.25) is about 100–200 nm, and the Cu-Bi bimetallic distribution is uniform. The particle sizes of Bi and Cu are 200 nm and 50–100 nm, respectively. Then we measured the element distribution using SEM-EDX. As shown in the mapping spectrum, Cu and Bi elements were uniformly distributed on the catalyst surfaces (Figures 2B,C; Supplementary Figure S2). Through SEM-EDX and XPS analysis, the element ratio of our prepared Cu-Bi was 0.8:0.2 (Supplementary Figure S3).

**FIGURE 3:**
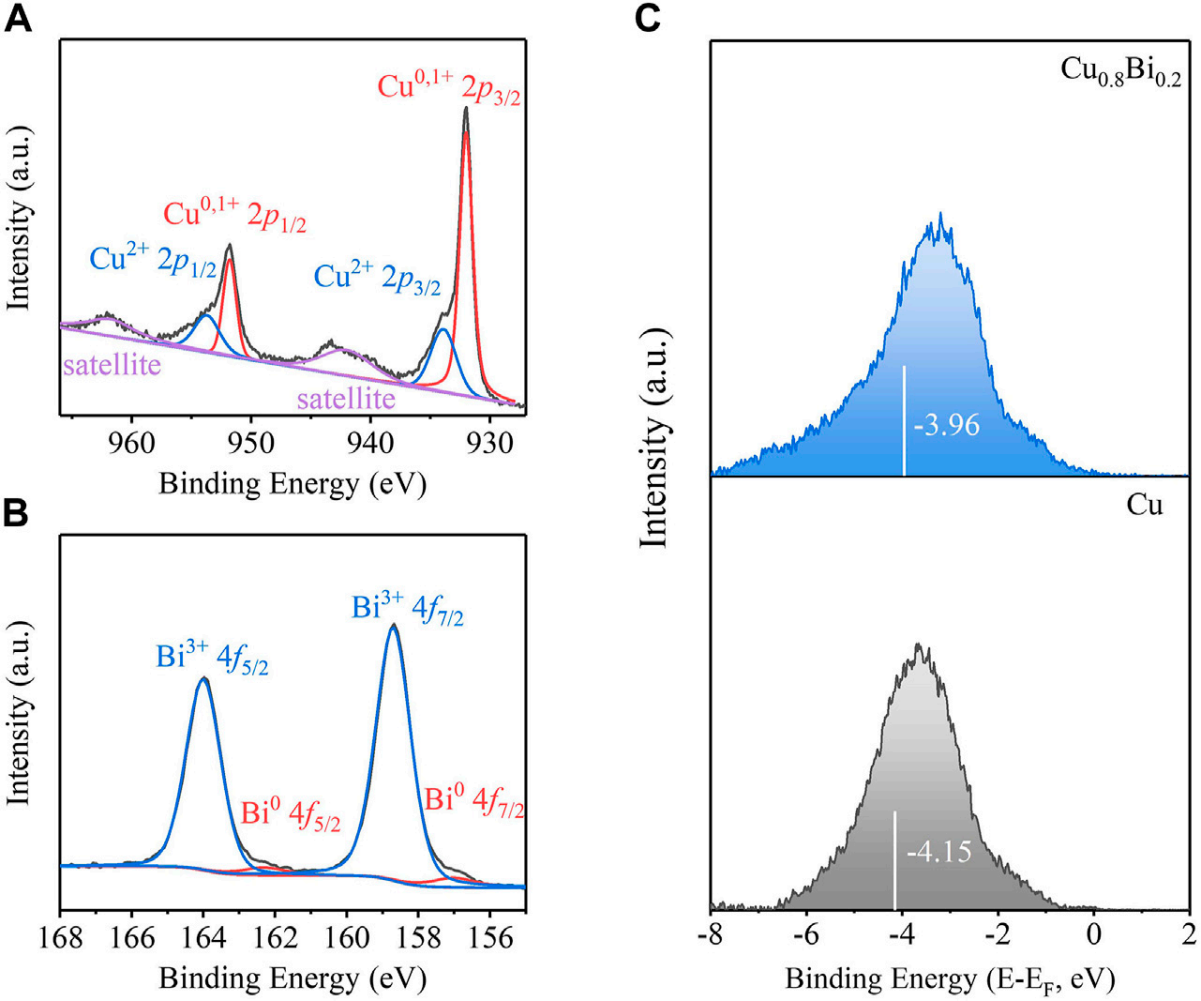
**(A)** Cu 2p XPS spectra of Cu_0.8_Bi_0.2_. **(B)** Bi 4f XPS spectra of Cu_0.8_Bi_0.2_. **(C)** Surface valence band photoemission spectra of Cu and Cu_0.8_Bi_0.2_. The white bar in **(C)** highlights the *d*-band center of various materials.

The chemical states of the synthesized Cu, Cu_0.8_Bi_0.2_, and Bi catalysts were studied by the high-resolution XPS spectra of Bi 4f and Cu 2p, respectively (Figures 3A,B). We observed that the strong peaks at 158.7 and 164.0 eV corresponded to Bi^3+^ 4f_7/2_ and Bi^3+^ 4f_5/2_, and the peaks at 157.0 eV, 162.3 eV corresponded to Bi^0^ 4f_7/2_ and Bi^0^ 4f_5/2_. From the high-resolution XPS spectrum of Cu 2p, the peaks at 932.0 and 951.8 eV corresponded to Cu^0, 1+^, while the peaks at 933.9 and 953.6 eV were consistent with Cu^2+^. It should be explained that both Cu and Bi are easily oxidized in air, the positively charged Bi^3+^ and Cu^2+^ are detected, probably because the oxidation occurred during the storage of the sample in air. Compared with Cu, the d-band center of Cu_0.8_Bi_0.2_ moved more positively after the introduction of Bi species (Figure 3C). As reported, the positively shifted d-band center likely increases the electron donation from the catalysts to the adsorbed *OCHO intermediate, strengthening the *OCHO surface binding (Xin et al., 2014; Zhang et al., 2018). This is consistent with our electrochemical CO_2_R test that Cu-Bi catalysts show improved performance.

**FIGURE 4:**
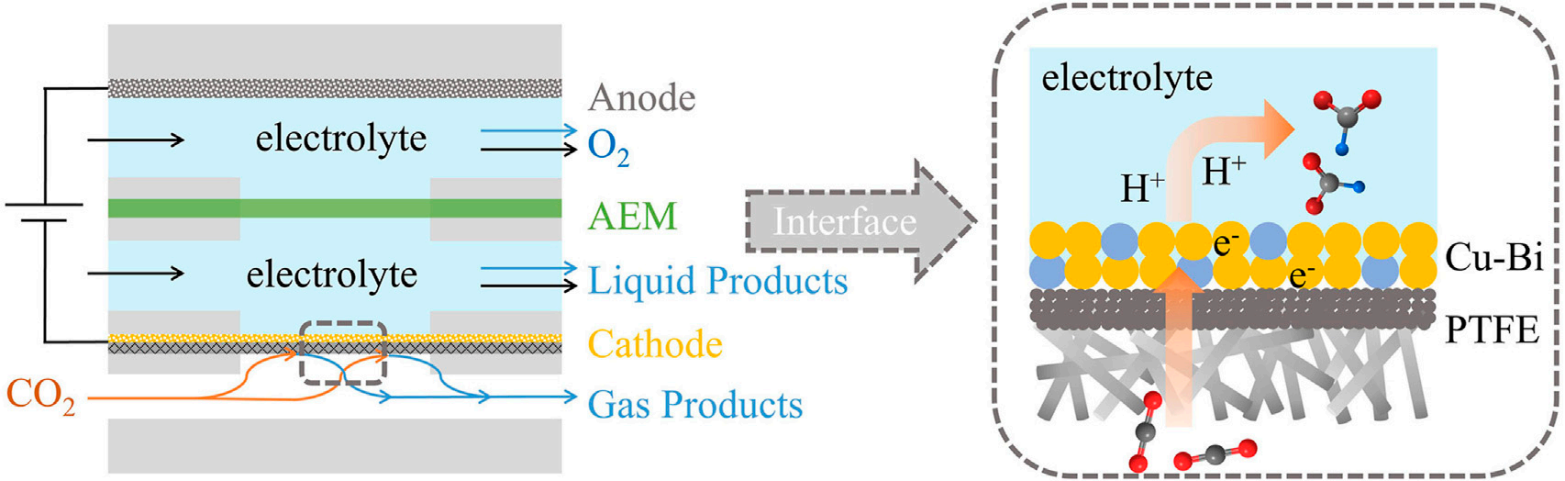
Schematic of Cu-Bi electrocatalyst on PTFE for electroreduction of CO_2_ in a flow cell.

**FIGURE 5:**
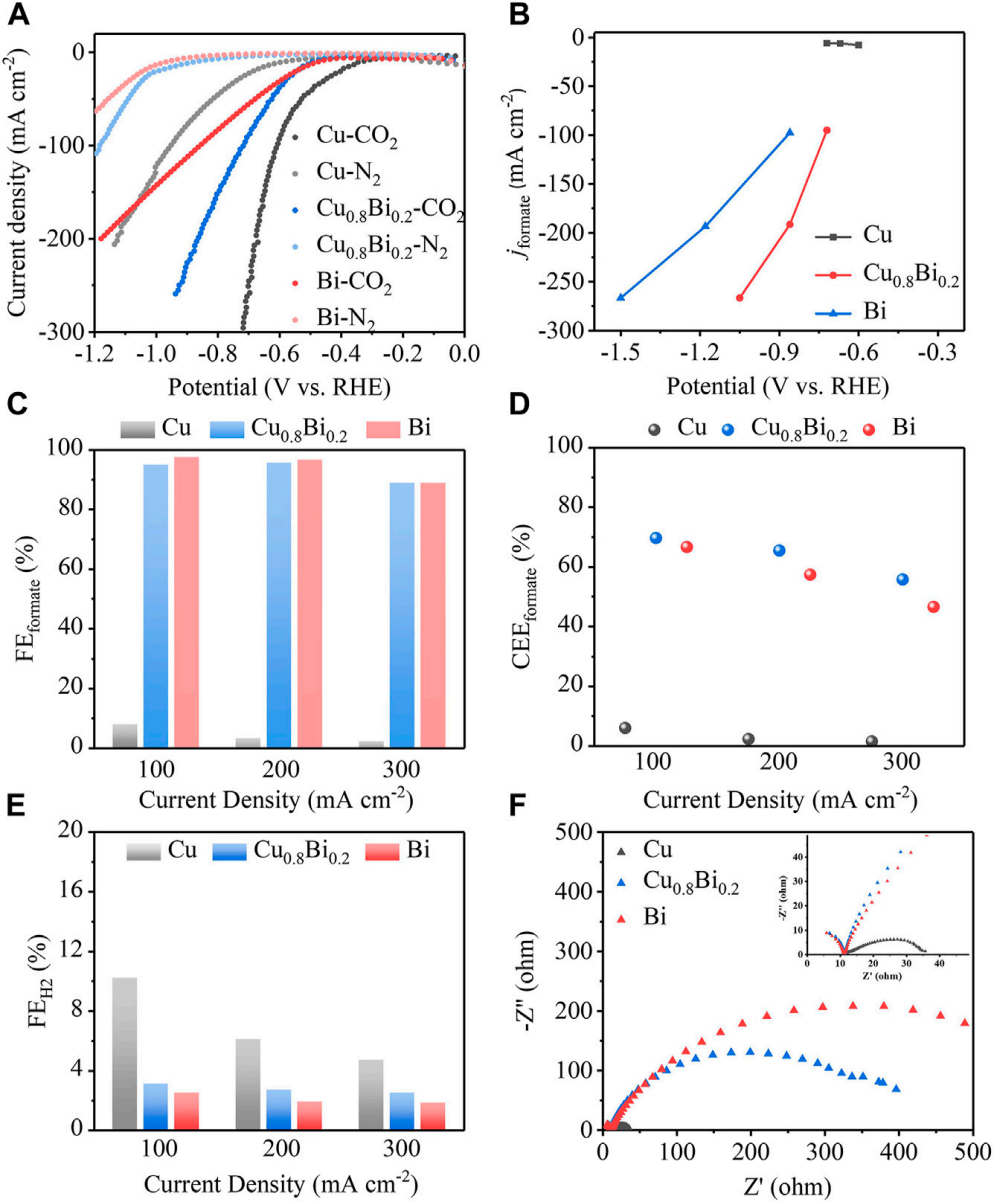
**(A)** The LSV curves under CO_2_ or N_2_ conditions of Cu_0.8_Bi_0.2_, Cu, and Bi. **(B)** The particular current density of formate of Cu_0.8_Bi_0.2_, Cu, and Bi. **(C,E)** The faradaic efficiency of formate and H_2_ of Cu_0.8_Bi_0.2_, Cu, and Bi at current densities of 100, 200, and 300 mA cm^−2^. **(D)** The cathode energy efficiency of formate at current densities of Cu_0.8_Bi_0.2_, Cu, and Bi of 100, 200, and 300 mA cm^−2^. **(F)** The EIS curve of Cu, Cu_0.8_Bi_0.2_, and Bi.

The CO_2_R electrochemical performance of the Cu_*1-x*_Bi_*x*_ (*x* = 0.1, 0.2, 0.25), pure Cu, and pure Bi catalysts were tested in a flow cell with a three-electrode system (Figure 4). The anode was Ni, the reference electrode was an Ag/AgCl electrode, and the electrolyte was 1 M KOH solution (pH = 14). From the linear sweep voltammetry (LSV), we can intuitively see that the reaction overpotential of Cu-Bi bimetal was reduced significantly (Figure 5A; Supplementary Figure S4). As shown in Figure 3B, the overpotential (520 mV) of the Cu_0.8_Bi_0.2_ catalyst is significantly smaller than that of the Bi catalyst (660 mV) at the same current density (100 mA cm^−2^). The overpotential for Cu is the best (400 mV), but the product selectivity is poor (Figure 5B). The cathodic current density on Cu, Cu_0.8_Bi_0.2_, and Bi electrodes was largely reduced when N_2_ was passing through (Figure 5A). In the presence of N_2_, the current density started to increase slowly at −0.70 V vs. RHE, which was mainly caused by the hydrogen evolution reaction (HER). It is also clear that HER on the Cu electrode is worse than CO_2_R on the Bi electrode, indicating that the introduction of Bi into Cu can suppress HER In the presence of CO_2_, which is more beneficial to CO_2_R.

To investigate the product selectivity of CO_2_R with Cu_0.8_Bi_0.2_, Cu, and Bi catalysts, chronopotentiometry tests were performed at current densities of 100, 200, and 300 mA cm^−2^, respectively (Supplementary Figure S5). The products were quantitatively analyzed by gas chromatography (GC), ion chromatography (IC), and nuclear magnetic resonance (NMR). As shown in Figures 5C-E, and Supplementary Figure S6, the Cu_0.8_Bi_0.2_ catalyst achieved over 90% selectivity to formate at all current densities, in particular, the selectivity for formate reached 95% at a current density of 100 mA cm^−2^, with a cathode energy efficiency reaching about 70%. Although the single-metal Bi catalyst has a formate selectivity close to that of Cu_0.8_Bi_0.2_, its cathode energy efficiency is much lower than that of Cu_0.8_Bi_0.2_ at all current densities due to its high reaction potential. In the case of the Cu catalyst, the formate selectivity is very low. Considering both the selective and energy efficiency, Cu_0.8_Bi_0.2_ outperforms Bi and Cu in the electroreduction of CO_2_ to formate.

As shown in Figure 5E, we found that the introduction of Bi into Cu can significantly reduce HER, which is consistent with the LSV results under N_2_ environment. To explain the reason for the excellent performance of Cu_0.8_Bi_0.2_ catalysts, we performed EIS tests on the three samples. As is shown in Figure 5F, Cu_0.8_Bi_0.2_ shows a smaller semicircle diameter than Bi in the impedance spectrum, suggesting that the charge transfer resistance of Cu_0.8_Bi_0.2_ is lower than that of Bi, ensuring a faster electron transfer during the reaction. The conductivity of Cu is 58.13953 S/m, and that of Bi is 0.95238 S/m. The conductivity of Cu is significantly better than that of Bi. Therefore, we analyze that the loading of Cu metal increases the conductivity of the material and reduces the charge transfer resistance, also the HER is greatly suppressed in the presence of CO_2_, as a result, Cu_0.8_Bi_0.2_ can reduce the reaction overpotential and maintain high CO_2_R catalytic activity and selectivity.

**FIGURE 6:**
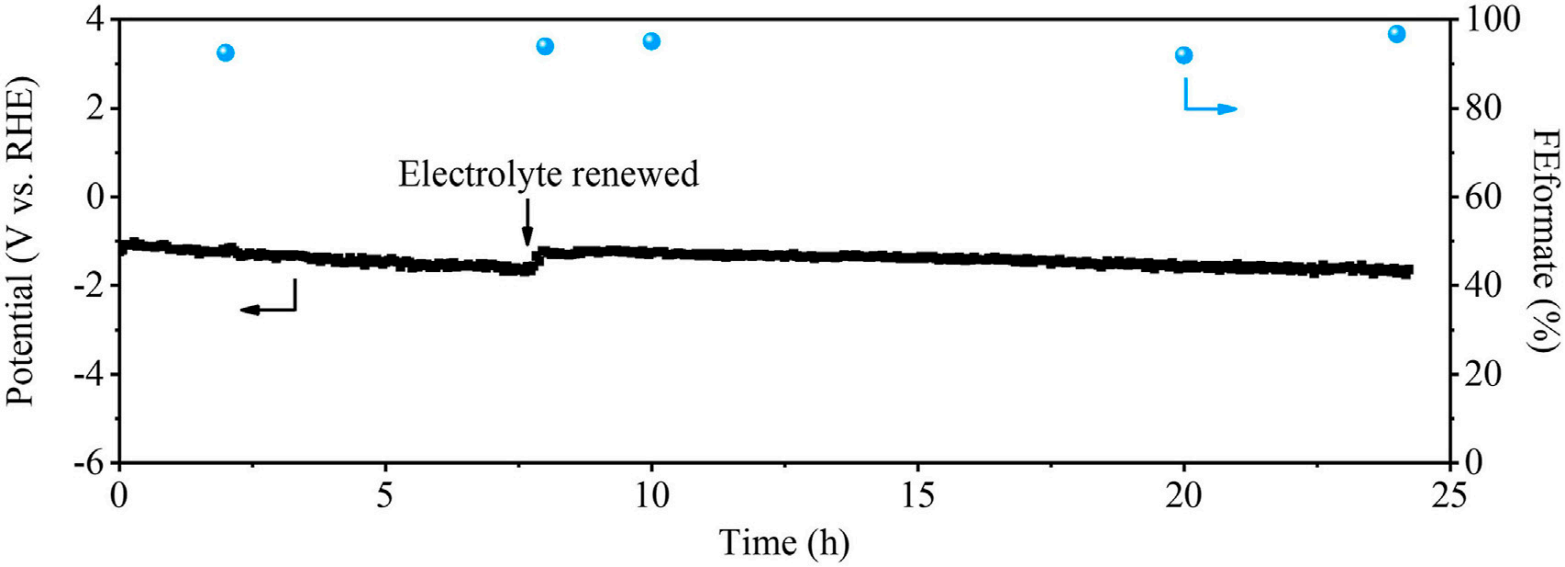
The stability test of Cu_0.8_Bi_0.2_ in 1 M KOH at 200 mA cm^−2^.

To verify the stability of the Cu_0.8_Bi_0.2_ catalyst, we performed the stability test at a current density of 200 mA cm^−2^, and SEM characterization of the reacted sample was performed. It can be seen from Figure 6 that during the reaction process of the 24 h CO_2_ reduction, the reaction potential did not change significantly, and the selectivity of HCOO^−^ was maintained above 90%. We also carried out SEM, SEM-EDX, and XRD analyses of the sample after 24 h of reaction. From Supplementary Figure S7, we can observe that the morphology and structure of the Cu_0.8_Bi_0.2_ bimetallic catalyst did not change after the reaction. The XRD characterizations also showed that the catalyst did not change significantly after the reaction, indicating that good stability with Cu_0.8_Bi_0.2_ for CO_2_R.

## Conclusion

In conclusion, we developed a simple, controllable, and large-area preparation method for the synthesis of Cu_0.8_Bi_0.2_ catalysts. Cu_0.8_Bi_0.2_ exhibited excellent formate selectivity and cathode energy efficiency under all current densities. Specifically, it exhibited a formate selectivity of 95% and a cathode energy efficiency of 70% at a potential of −0.72 V vs. reversible hydrogen electrode and maintained the CO_2_R durability for over 24 h at a current density of 200 mA cm^−2^. The excellent catalytic performance of Cu_0.8_Bi_0.2_ is attributed to the following factors: 1) CuBi alloy likely has a favorable work function to improve CO_2_ adsorption for formate production; 2) CuBi alloy improves electron transport. We expect that the bimetal Cu_0.8_Bi_0.2_ electrocatalyst may offer a material foundation for the improved catalytic CO_2_R to formate conversion.

## Data Availability

The original contributions presented in the study are included in the article/Supplementary Material, further inquiries can be directed to the corresponding author.
