# Supplementary material for: Bimetallic Cu-Bi catalysts for efficient electroreduction of CO2 to formate
Source: Front Chem. 2022 Oct 3;10:983778. doi: 10.3389/fchem.2022.983778 (PMC9573945; doi:10.3389/fchem.2022.983778)
Supplement: Supplementary file 1 [file DataSheet1.PDF]

Supplementary information

Bimetallic Cu-Bi catalysts for efficient electroreduction of CO_2 to formate

Le Li[‡,1], *Xuan Jin*[‡,1], *Xiaohan Yu*[1], *Miao Zhong*[*,1]

[1]College of Engineering and Applied Sciences, National Laboratory of Solid State Microstructures, Collaborative Innovation Center of Advanced Microstructure, Jiangsu Key Laboratory of Artificial Functional Materials, Nanjing University, 210093, China.

‡ These authors contributed equally to this work.

* Correspondence: Miao Zhong (miaozhong@nju.edu.cn)

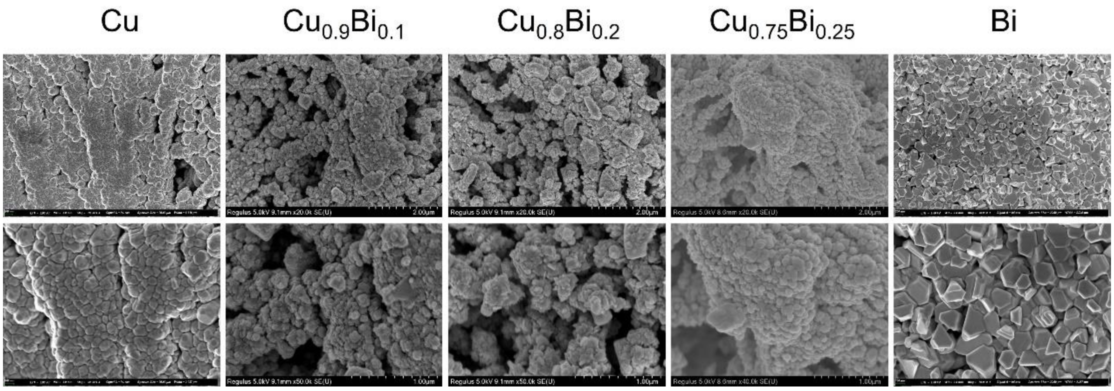

Fig. S1. SEM images of the prepared Cu, $Cu_{0.9}Bi_{0.1}$, $Cu_{0.8}Bi_{0.2}$, $Cu_{0.75}Bi_{0.25}$, and Bi.

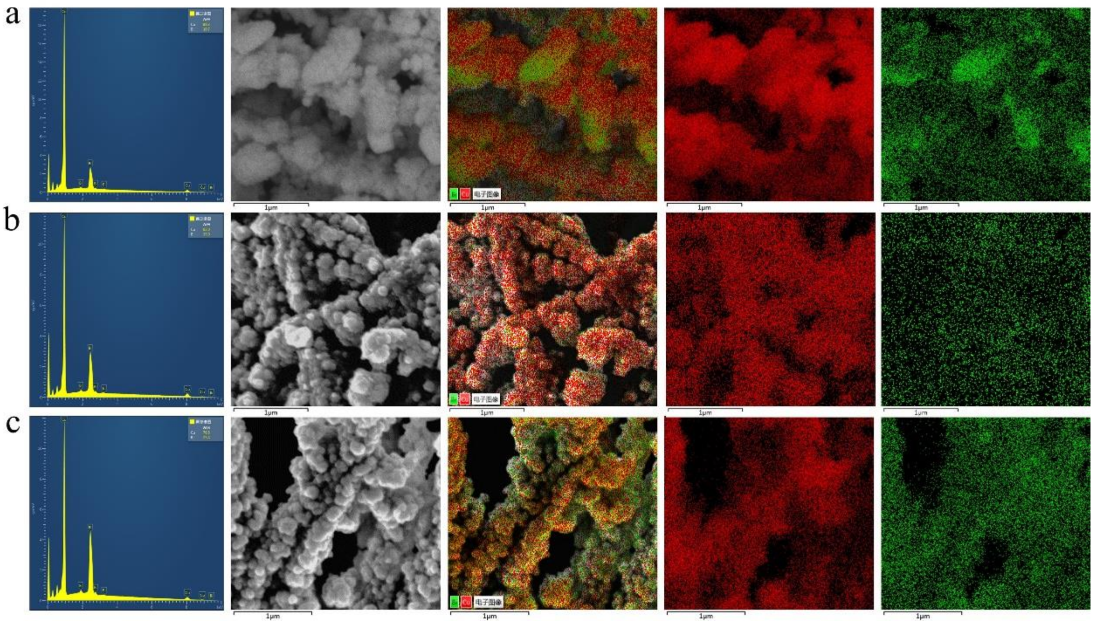

Fig. S2. EDX spectrum and EDX elemental mapping results in SEM of a. $Cu_{0.9}Bi_{0.1}$. b. $Cu_{0.8}Bi_{0.2}$. c. $Cu_{0.75}Bi_{0.25}$.

	SEM-EDX	XPS
	Atomic %	Atomic %
Cu	81	82
Bi	19	18

Fig. S3. The molar concentration of Cu and Bi in the $Cu_{0.8}Bi_{0.2}$ obtained by SEM-EDX and XPS analyses.

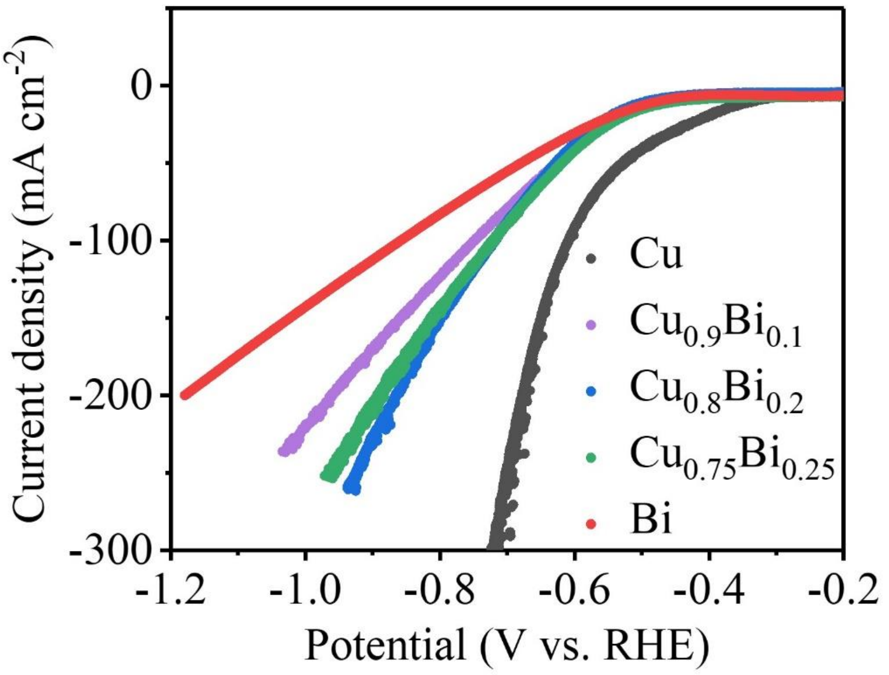

Fig. S4. Linear sweep voltammetry (LSV) curves of the $Cu_{1-x}Bi_x$ (x=0.1, 0.2, 0.25), Cu, and Bi catalysts in 1 M KOH electrolyte.

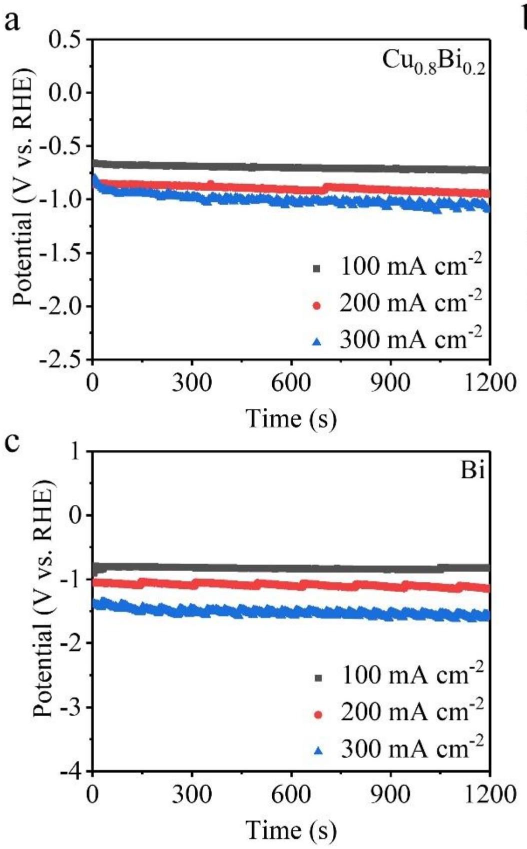

Fig. S5. The CO_2-reduction chronopotentiometry curve of the a. $Cu_{0.8}Bi_{0.2}$, b. Cu, and c. Bi catalysts in 1 M KOH electrolyte at 100, 200, 300 mA cm^{-2}.

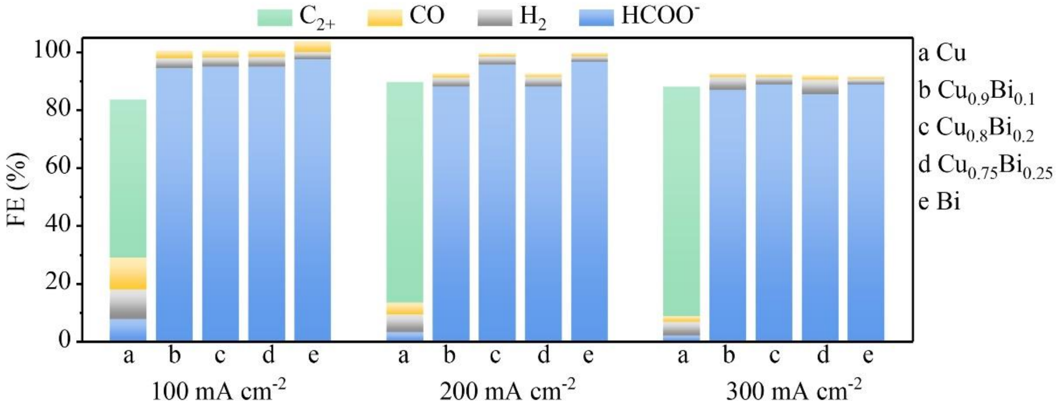

Fig. S6. The $HCOO^-$, H_2, CO, and C_{2+} FEs under different current densities of $Cu_{1-x}Bi_x$ (x=0.1, 0.2, 0.25), Cu, and Bi catalysts in 1 M KOH electrolyte.

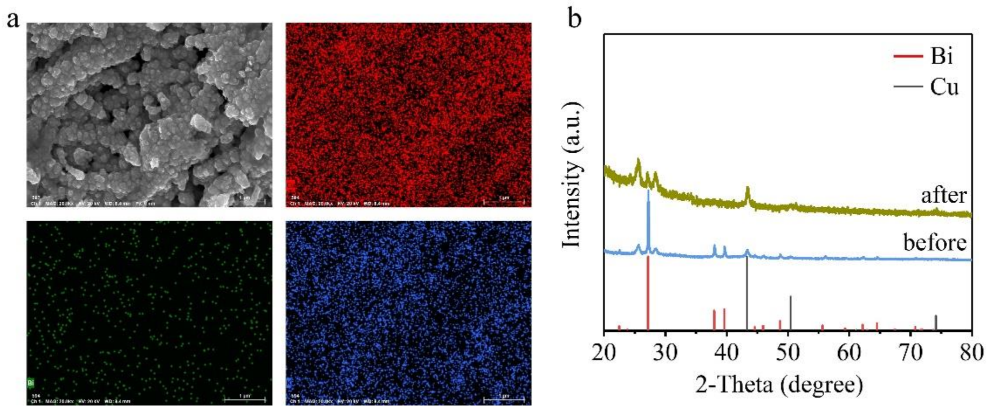

Fig. S7. a. SEM-EDX elemental mapping results. b. XRD data of the $Cu_{0.8}Bi_{0.2}$ catalyst over 24 h of reaction.
